# 

**Published:** 1897-06-05

**Authors:** 


### EARLY EDITION.

**Notices of Births, Deaths, and Marriages** are inserted in this column at a charge of 2s. 6d. for an announcement not exceeding four lines.

**NOTICE TO CORRESPONDENTS.**
All MS., Letters, Books for Review, and other matters intended for the Editor should be addressed The Editor, "The Hospital," The Hospital Building, 28 & 29, Southampton Street, Strand, W.C.
The Editor cannot undertake to return rejected MS., even when accompanied by stamped directed envelope.

**Notice to Advertisers and Subscribers.**
All Advertisements, Orders for Copies of The Hospital, and Business Communications should be addressed to **THE MANAGER, (Not to The Editor),** The Hospital, The Hospital Building, 28 & 29, Southampton Street, Strand, London, W.C.
The Cost of Subscription, post free, is as follows:—Per week, 2½d.; per quarter, 2s. 8d.; per half-year, 5s. 3d.; per annum, 10s. 6d. Foreign:—Per annum, 15s. 2d.; payable, in all cases, in advance.

### BOOKS RECEIVED.

ISBISTER AND CO.

"Marie Hilton: Her Life and Work." By J. Deane Hilton.

CHARLES GRIFFIN AND CO.

"Year-Book of Scientific and Learned Societies."

P. BLAKISTON, SON, AND CO.

"Retinoscopy." By James Thorington, M.D.

JAMES BOWDEN.

"The White Slaves of England." By R. H. Sherar

### The Student of Medicine.

W. Allport, M.B., B.S.Lond., M R.C.S.Eng., L.R.C.P.Lond., appointed House Surgeon to the Queen's Hospital, Birmingham. W. B. Bennet, M.R.C.S., L.R.C.P., appointed House Surgeon to the Liverpool Royal Infirmary. R. M. Cowie, M.R.C.S, L.R.C.P., appointed House Surgeon to King's College Hospital, Lincoln's Inn Fields. C. C. J. Erhardt, M.R.C.S, L.R.C P., appointed House Accoucheur to King's College Hospital, Lincoln's Inn Fields. G. Flux, M.D Brux., M.R.C.S., L.R.C.P., L.S.A., appointed Anæsthetist to the British Lying-in Hospital. A. R. Galloway, M.A, M.B., C.M.Aberd., appointed Surgeon to the Aberdeen Ophthalmic Institution. H. B. Gladstone, M.B., M.S.Edin., appointed House Physician to the Bradford Infirmary. T. C. Green, M.D.Brux., L C.R.P.andS Edin., appointed Junior Assistant Medical Officer at the County Asylum, Bracebridge, Lincoln. F. R. Greenwood, M.R.C.S.Eng., L.R.C.P.Lond., appointed House Physician to the Queen's Hospital, Birmingham. F. J. Hasslacher, M.R.C.S., L R C.P., appointed House Surgeon to King's College Hospital, Lincoln's Inn Fields. J. Hay, M.B., Ch.B.Vict., M.R.C.S., L.R.C.P., appointed a House Physician to the Liverpool Royal Infirmary. F. H. Jacob, M.R.C.S., L.R.C.P., appointed House Surgeon to King's College Hospital, Lincoln's Inn Fields. M. B. James, M.B., B.Ch., appointed Medical Officer to the Highgate Workhouse and Infirmary. H. F. Jeffery, M.B., Ch.B.Vict., appointed Senior House Surgeon to the Bury Infirmary. T. H. Kellock, M.A., M.D., B.C.Cantab, F.R.C S., L.R.C.P., appointed Assistant Surgeon to the Middlesex Hospital. G. G. Lawson, M.B., Ch.B.Vict., appointed House Physician to the Liverpool Royal Infirmary. F. S. Lynch, M.R.C.S., L.R.C.P., appointed Assistant House Physician to King's College Hospital, Lincoln's Inn Fields. J. S. MacDonald, L.R.C.S., L.R.C.P.Edin., etc., appointed House Physician to Liverpool Royal Infirmary. J. R. MacMahon, M.B., C M., M.R.C.S., M.R.C.P., appointed Clinical Assistant at St. John's Hospital for Diseases of the Skin, London. F. E. Marshall, M.B., Ch.B.Vict., L.S A., appointed a House Surgeon to the Liverpool Royal Infirmary. T. H. E. Meggs, M.R.C.S., L.R.C.P., appointed Medical Officer to the Eton Union Workhouse and the Upton-cum Chalvey District. M. Monier-Williams, M.R.C.S., M.R.C P, appointed Clinical Assistant at St. John's Hospital for Diseases of the Skin, London. S. Paget, M A.Oxon, F.R.C.S., appointed Aural Surgeon to the Middlesex Hospital. A. Price, M.R C.S.Eng., L.S.A., appointed Medical Officer to Walton Gaol, Liverpool. Dr. Rutherford, appointed Assistant Surgeon to the Royal Infirmary, Newcastle-on-Tyne. E. S Smith, M.R.C.S.Eng., L R.C.P.Lond., appointed Obstetric and Ophthalmic House Surgeon to the Queen's Hospital, Birmingham. G. V. Smith, M.B.Lond, appointed House Physician to King's College Hospital, Lincoln's Inn Fields. J. E. Smith, M.B., Ch.B.Vict., appointed House Surgeon to the Lock Hospital, and Assistant in Eye and Throat and Ear Department of the Liverpool Royal Infirmary. M. F. Squire, M.B., B.S., M.R.C.S.Eng., L.R.C.P., Lond., appointed Assistant Medical Officer to the Workhouse of the Paddington Union.

